# Few Effects of Far Transfer of Working Memory Training in ADHD: A Randomized Controlled Trial

**DOI:** 10.1371/journal.pone.0075660

**Published:** 2013-10-04

**Authors:** Jens Egeland, Anne Kristine Aarlien, Brit-Kari Saunes

**Affiliations:** 1 Division of Mental Health & Addiction, Vestfold Hospital Trust, Tønsberg, Norway; 2 Department of Psychology, University of Oslo, Oslo, Norway; 3 Division of Child and Adolescent, Telemark Hospital Trust, Skien, Norway; Catholic University of Sacred Heart of Rome, Italy

## Abstract

**Objective:**

Studies have shown that children with ADHD profit from working memory training, although few studies have investigated transfer effects comprehensively. The current Randomized Controlled Trial analyzes transfer to other neuropsychological (NP) domains, academic performance and everyday functioning at home and school.

**Method:**

Sixty-seven children with ADHD were randomized into a control group or a training group. The training group underwent Cogmed’s RoboMemo program. All participants were assessed pre-training, immediately after and eight months later with a battery of NP tests, measures of mathematical and reading skills, as well as rating scales filled out by parents and teachers.

**Results:**

There was a significant training effect in psychomotor speed, but not to any other NP measures. Reading and mathematics were improved. There were no training induced changes in symptom rating scales either at home or at school. The increased reading scores remained significant eight months later.

**Conclusion:**

The study is the most comprehensive study of transfer effects to date, and with mixed results compared to previous research. More research is needed regarding how to improve the training program and the conditions and thresholds for successful training.

**Trial Registration:**

Controlled-Trials.com ISRCTN19133620

## Introduction

Impaired working memory (WM) is characteristic of a multitude, or even most, neuropsychiatric [1] and developmental disorders [2]. A meta-analysis of studies of WM performance in ADHD [3] showed impairments ranging from half to more than one standard deviation, depending on whether tasks demanded information manipulation or merely storage. There are several models of WM [4], [5]. Common to them is that WM operates on a limited time period after information is being presented enabling the person to hold the information ‘online’ for the time needed to process it. Simple storage is equivalent to short term memory or attention span, either within the visual of auditory modality, while manipulation involves higher level executive functions. Typical tasks measuring storage is visual or auditory span tasks, measuring the extent of information the person can grasp without rehearsing. Manipulation tests can differ from *simple* manipulation involved in reversing a sequence of numbers held in simple storage, to more *complex* manipulation involved for example in reorganizing both numbers and letter sequences [6]. In the multimodal model of Baddeley [4], simple storage is part of the WM model, although differentiated from the Central Executive. Other researchers have found evidence of the distinction of short term memory vs. working memory [7], and reserve the WM construct for manipulation only. The working memory training regime tested here is based on Baddeley’s model incorporating both simple storage in the short-term memory sense as well as manipulation, into the model.

Several studies have reported that WM capacity in ADHD [8], [9] and other disorders [8], [10] can be improved by training on specially designed computer-based training programs. The producer of Cogmed-program RoboMemo claims that training has been implemented in more than 800 schools in Sweden, where the program was developed [11]. Pearson Assessment is marketing the program in the USA, and promoting it as an effective treatment of WM deficit in several disorders in addition to ADHD. On the other hand, recent studies have harshly criticized these positive claims [12], [13] initiating a debate about the theoretical and empirical basis for a possible treatment effect [14], [15].

There are several reasons for being enthusiastic about WM training: Whereas medication effects depend on continued use, WM training can potentially cure the deficit in the sense that the effect lasts beyond the training period. How long it lasts is not clear, since no study to date has had follow up more than 6 months after completed training [16], [17].

Some parents do not want their children to use stimulant medication. In some cases medication is terminated due to side effects or lack of treatment effect on everyday behavior. Although considered an effective drug in reducing behavioral symptoms of ADHD, another reason for exploring the possibility of training WM is that methylphenidate (MPH), may not have the equivalent effect on working memory as on other aspects of executive function (EF). Some studies have shown no effect on WM while attention in general is improved [18], [19]. A metastudy by Pietrzak and colleagues [20] showed that only half of published studies of MPH-treatment showed effects on the manipulation element of WM. The authors discuss the possibility that the modulation of dopamine turnover affects WM less, possibly because WM requires simultaneous application of multiple cognitive processes exceeding those modulated by MPH.

While WM training represents a longed-for non-pharmacological treatment of cognitive symptoms of ADHD, it is nevertheless important to assess the transfer effects critically. Reports from new and experimental treatments will typically suffer from a publication bias. Negative findings are not relevant since the treatment is not broadly applied. Shipstead, Redick and Engle [21] present a list of factors that can contaminate the validity of training experiments. They claim that the effects on measures of WM are sufficiently documented, while transfer effects are not. They point out that results on single tests cannot be interpreted as an increase in a *function*, but that multiple measures of the same construct must converge to conclude about a transfer effect. In addition, if several studies conclude on transfer effects within different domains, the overall picture may be more negative.

The meta-analysis of Melby-Lervåg and Hulme [12] offers an excellent overview of previous research. The authors conclude that WM training programs appear to produce short-term, specific training effects that do not generalize to tasks that are different in content, but that still are WM demanding. However, most individual studies have so far found positive effects. Below, we refer to the most influential papers so far.

Using the same training program as the present study, Klingberg and colleagues [8], [9] report increased visual reasoning, while Holmes et al. [17] found increased mathematical reasoning. Klingberg [9] reports increased parent reported attention, with no equivalent effect rated by teachers. Some positive findings are difficult to understand in terms of increased WM. One such effect is related to non-verbal IQ that was found to be increased immediately after training in the Klingberg studies [8], [9]. Studies applying the WASI [22] have not found such transfer effects [17], [23]. The increase in Raven’s Matrices might be a specific effect of training in solving visual tasks not expected to transfer to everyday functioning. It might also be an unspecific effect of increased motivation. But then again, other performance measures should also increase. Løhaugen et al. [10] found that memory was increased after training in both healthy and extreme low-birth weight adolescents.

Regarding academic performance, the few existing studies are inconsistent. Dahlin [16] reports an increase in reading comprehension, while Holmes et al. [23] found an increase in mathematic problem-solving, but no increase in reading comprehension. Applying another computerized WM training program, Loosli and colleagues [24] found modest increases in word and text-reading, but not in nonsense-word decoding.

Beck, Hanson and Puffenberger [25] performed the largest and most comprehensive study to date of behavioral transfer effects rated by parents and teachers. Their main findings were better symptom ratings at home, but minimal change at school.

At this point in the research on WM training, it is necessary to assess possible effects broadly, reporting from different functional domains and different levels of transfer simultaneously and comprehensively. In the present study, the participants have been assessed with a broad range of 1) working memory measures, 2) tests of l (NP) performance, 3) tests of skills in reading and mathematics, as well as behavior rating scales by 4) parents and 5) teachers.

The direct effects on measures of WM are in the process of being published elsewhere. In short, this study reported a consistent increase in the training group and a successful transfer from training to tasks similar to the ones that are trained. However, it remains unclear how this finding should be interpreted. It could merely be a task-specific increase, or it could represent an increase in WM as a cognitive function. In untrained subjects, tasks measuring auditory or visual span forward and backward or manipulation of letters and numbers simultaneously in WM are considered valid measures of working memory. However, if increased performance after training is limited to handling numbers or remembering the order of a number of visual objects without effect on other cognitive functions or daily life, we would not interpret the increase as an increase in the cognitive function of working memory.

Thus, in this analysis of far transfer effects we ask whether the increased WM performance transfers 1) to other NP functional domains, i.e. selective attention, sustained attention or learning capacity; (2) to academic skills such as mathematics and reading ability, and (3) whether parents and teachers rate the training children as less symptomatic with regard to a) working memory, b) attention in general, and c) ADHD symptoms. We investigate the presence of any far transfer effects both immediately after training and 8 months post-training.

## Materials and Methods

### Participants

**Figure 1:**
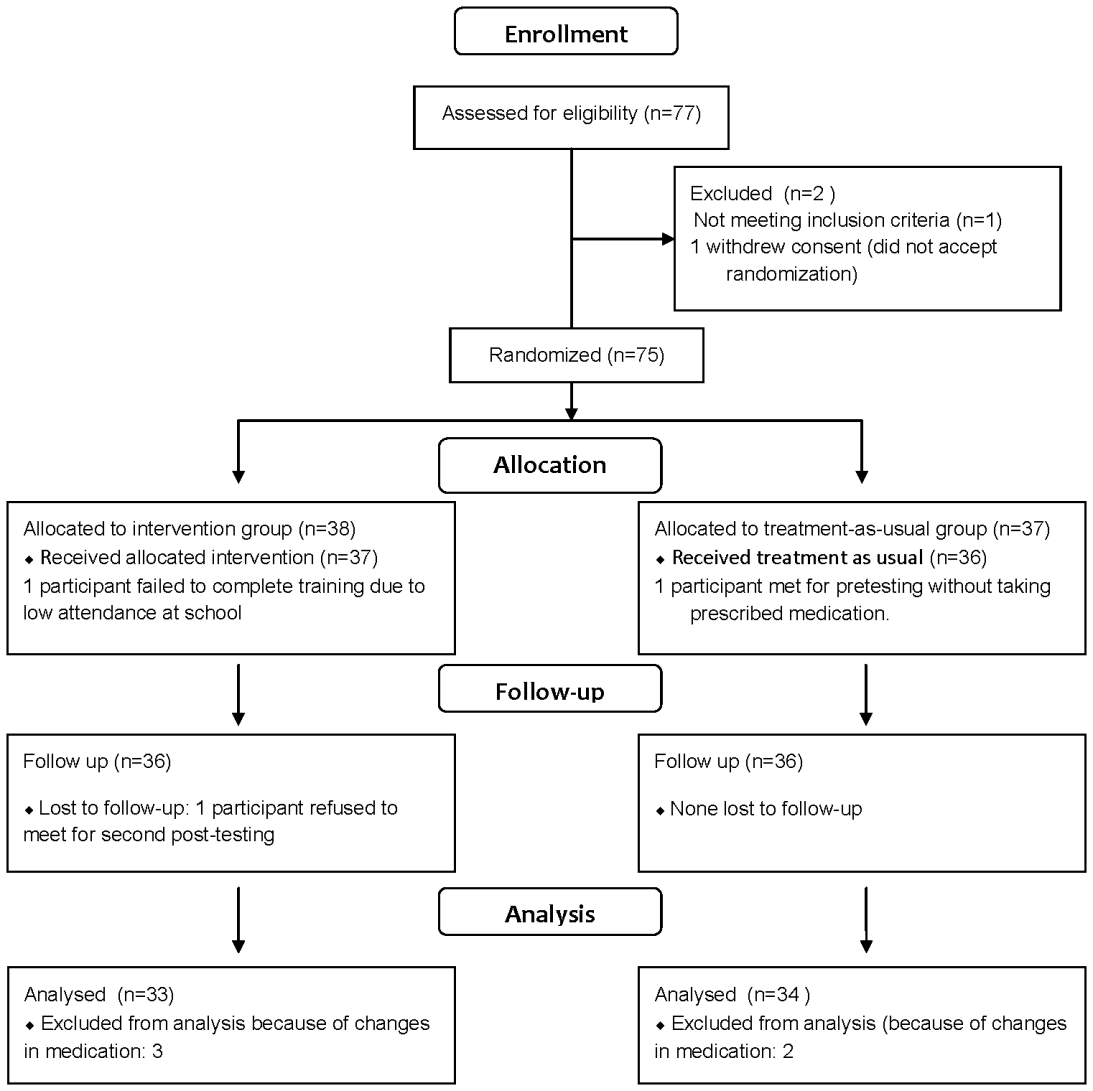
Participant Flow Diagram.

The flow of participants in the study is presented in Figure 1. The protocol for this trial and supporting Consort checklist are available as supporting information, see Checklist S1 and Protocol S1 and S2.

In short, 72 of 77 recruited children aged 10 to 12 years completed the study. Five children were omitted from the present analyses due to changes in medication during the project period. The presented analyses are based on the remaining 67 subjects. All had a confirmed diagnosis of F-90 ICD-10 Hyperkinetic Disorder [26], equivalent to the DSM-IV diagnosis of ADHD combined type. All were in treatment for ADHD within the Departments for Child and Adolescent Psychiatry in Vestfold or Telemark Hospitals, Norway. Exclusion criteria were IQ below 70, or a comorbid diagnosis of Pervasive Developmental Disorders, Tourette’s Disorder, evidence of psychosis or Bipolar Disorder and Conduct Disorder.

Forty-one of the final participants were medicated with MPH in the same dosage throughout the study, whereas five used atomoxetine. One patient used risperidone as well. Due to a negative attitude against medication among parents or as a result of medication being discontinued, 21 participants did not use medication at the time of inclusion.

Forty-nine boys and 18 girls participated. Mean age was 10.4 (s.d. 0.7) and mean IQ was 94 (s.d. 12). There were no differences between the groups with regard to these characteristics, nor with regard to the education level of their parents.

### Trial Design

The study was a randomized controlled trial. The half of the participants drawn to compose the control group was offered the possibility to train after the completion of the study. The experimental intervention is described below. Except for this intervention both groups received treatment as usual. The experimental design included a pretest immediately before the start of the training period, a post-test at the conclusion of the training period and follow-up testing. The original plans called for follow-up testing to take place six months after conclusion of the training period, but due to unforeseen delays in completing the training program due to a flu epidemic, the actual follow-up testing took place eight months after conclusion of the training period. Participants meeting the inclusion criteria were matched on gender and medication status, and randomized by drawing numbers corresponding to ID numbers to either training or control group by a staff member not involved in training or testing. Staff members responsible for testing were not involved in conducting the training sessions for the intervention group, which took place at participants’ schools administered by a teacher or other person designated by a school official. Training sessions took place at the participant’s school during regular school hours and all testing took place at the Departments for Child and Adolescent Psychiatry in Vestfold or Telemark Hospital Trusts, Norway. The study was approved 18.06.2009 by the Regional Committees for Medical and Health Research Ethics (REC South East). Recruitment and enrollment began in 01.08.2009 and follow-up testing was completed 31.12.2010. All parents, as well as children over 12 years of age, had signed written consent forms prior to participation. This trial was prospectively registered with, and publicly available on, the website of the Norwegian Ethics Committee in 2009: https://helseforskning.etikkom.no/ikbViewer/page/prosjekterirek/prosjektregister?_ikbLanguageCode=us&p_dim=34977&9F508B87E7D8620DE040F28156A418DC.p_search_id=26503.

This study is also registered as ISRCTN19133620 (www.controlled-trials.com).

### WM Training Intervention

The WM training consisted of Cogmed’s RoboMemo program performed on a daily basis at school for 5–7 weeks. The program lasts for 30–45 minutes and consists of 13 adaptive exercises selected from an algorithm that continually increased or decreased the difficulty level of each exercise according to the child’s performance. Thus, the participants were systematically working on tasks taxing their WM capacity. The training regime includes three letter span tasks (all forward condition), three digit span tasks (one forward condition, two backward conditions), and seven visuospatial tasks (all forward sequenced), including static visuospatial tasks (one 2D visuospatial task, one 3D visuospatial task), and two dynamic visuospatial tasks, in which students recall the positions of rotated or moving objects. Nine of the tasks are presented purely in visual format, and four are delivered with an auditive input. Eleven of the tasks are forward sequenced, while only two are reverse order tasks.Every five days, new training tasks were introduced to replace the earlier tasks. In every session, the participant trains both storage and manipulation visually and verbally. Nine tasks were visual, while four were auditory as well.

The participant received daily verbal and visual feedback about increases in performance and personal records and was rewarded after training by being allowed to play the RoboRacing-computer game. Every fifth day the participant received an additional individualized reward. A teacher or assistant was present during training. Regarding training compliance, four subjects completed less than the planned number of 25 training sessions (3 had 24 sessions and one had 21). Average improvement index in the training program for the whole group was 24 (s.d. 8), slightly less (23) for those completing less than 25 training days. Four subjects discontinued training for about one week or more during the planned training period due to a flu epidemic. These days were compensated for by training additional days when they returned to school. Due to varying lengths of sick leave, one participant trained for 26 days and three trained for 30 days.

### Measures

The participants were examined with a wide range of tests and measures. As the project attempted to be more comprehensive than previous studies in assessing transfer effects, it was important not to single out selected measures for analysis. However, reporting on potentially more than one hundred between-group analyses would increase the risk of type I errors, as some effects could be accidentally significant. On the other hand, controlling statistically for multiple comparisons would require such a low p-level that nearly no effect could be statistically significant, increasing the risk of Type II errors. Thus, in adherence to the methodological critique of previous research by Shipstead et al. [21] we chose to reduce the number of comparisons by computing composite measures where relevant and possible. Below is a presentation of the measures applied and the computation of composite measures.

### NP Measures

#### Color Word (CW) and Trail Making (TMT) tests from the Delis-Kaplan Executive Function System (D-KEFS [27])

The CW-test is an extended version of the Stroop test [28] based on four tasks: Color Naming, Word Reading, Inhibition and Inhibition-Switching. The first two tasks are considered to tap semi-automatic and automatic processes, while the two Inhibition tasks demand controlled attention. TMT is an extended version of the Halstead-Reitan Trail Making Test [29]. Tasks 2 through 4 were administered. Tasks 2 and 3 tax simple visual search and are considered to measure simple processing speed, while Task 4, equivalent to TMT B in the original Halstead-Reitan version, is considered to measure divided attention.

The scaled scores of CW 1 and 2 and TMT 2 and 3 were averaged into a composite measure of Processing speed. The average scaled scores for speed and errors on CW 3 and 4 made up a measure of Controlled Attention, primarily taxing inhibition. TMT 4 measures a different aspect of controlled attention and is reported separately.

#### Conners’ Continuous Performance Test-II (CCPT-II: [30])

This is a high signal-to-noise continuous performance test (CPT) lasting 14 minutes and yielding 12 measures. Factor-analysis has shown that these can be reduced to four dimensions [31], [32]. Composite measures for each dimension were computed. *Focused attention* is the mean T-score of Omissions, Perseverations, Variability and Hit Reaction time Standard Error. *Hyperactivity-Impulsivity* is computed from the T-scores of Hit Reaction Time, Commission Errors and Response Style. *Sustained attention* is computed from Block Change and Block Change Standard Error, measuring the change in reaction time or increase in variability of reaction time as a function of time on task. *Vigilance* is computed from the Interstimulus-interval-change score as well as the Standard Error of ISI-change, measuring a possible fall in reaction time following longer interstimulus-intervals.

Two tests of memory were applied: *Children’s Auditory Verbal Learning Test-2* (CAVLT-2: [33]) and Benton Visual Retention Test, fifth edition. (BVRT) [34]. Three measures derived from the standard administration of CAVLT-2 are reported: *Level of Learning,* which is the number of correct responses to acquisition trial 3–5; *Free Delayed Recall* which is the number of correctly retrieved items after 30 minutes and *Recognition* which is the number of correctly identified items from a 32-item long recognition list.

In BVRT, the child is shown 10 designs, one at a time, and asked to reproduce each one as accurately as possible on a plain piece of paper from memory. The measure reported is the number of correctly reproduced designs. In listing this test as a memory test, we adhere to the manual and to a convention within in neuropsychology. As, there is no delay necessitating long term storage, it could in fact be considered a measure of WM.

### Academic Skills

#### Key Math [35]

Two subtests were applied: The Mental computation subtest consists of 18 verbally presented tasks with or without simultaneous visual information to be answered within 15 seconds (except for three tasks). The un-timed Problem-solving subtest involves 18 daily life math problems, where the subject has to decide on what part of the text presented is relevant and what procedures to apply. The scaled scores from both tests were averaged into a composite Mathematics score.

Reading ability was assessed with the computerized test battery *LOGOS* [36], based on the dual route-model of reading comprehension and word decoding. Decoding is analyzed phonologically and orthographically. The test yields 10 scores measuring different aspects of reading and text comprehension. Because they represent the end-point of reading training, Text Reading speed and Percent correct read are reported as single scores. These measures were not normally distributed, and could not be included in a composite score. Two composite scores representing Word Decoding Speed and Quality of Decoding, were also computed. These composites consisted of the average processing time and the average correctly processed single words under three conditions: phonologically based reading of meaningful and nonsense words as well as orthographic reading of single words.

### Rating Scales

*ADHD-Rating Scale IV* [37] was distributed to both parents and teachers. The scale yields separate measures for symptoms of inattention and hyperactivity/impulsivity as well as a total score. The Attention score is considered the most relevant measure of possible transfer effect of WM training and is reported together with the total score measuring possible overall symptom reduction.

*Strengths & Difficulties Questionnaire* [38], [39] is a measure of pro-social behavior and psychopathology of 3–16 year olds and was also completed by both parents and teachers. It yields separate measures for emotional symptoms, conduct problems, hyperactivity-inattention, peer problems and a prosocial scale. We report the Overall score computed from the four problem/symptom-scales, as well as the Impact score measuring the degree to which the problem behavior interferes with everyday functioning across different functional domains.

*Behavior Rating Inventory of Executive Function* (BRIEF: [40]) parent and teacher versions: The BRIEF is designed to assess executive functioning in home and school environments in children aged 5–18. The inventory yields eight subscales grouped into two indexes and one sum-score. We report the Metacognition index based on the Initiate, Working Memory, Plan/Organize, Organization of Material and Monitor-subscales. The Global Executive composite (GEC) is also reported. It is an overall measure based on all 8 scales, i.e. including the three scales constituting the Behavior Regulation index (BRI). The BRIEF has been proven useful in describing the details of EF function in ADHD and previously been used in assessing the effects of WM-training [25]. The Norwegian version is comparable to the original US version [41].

### Data Analysis

Demographic and clinical background information at baseline was analyzed with Analysis of Variance for continuous variables and Chi-square for categorical variables. Baseline levels for all dependent measures were compared between the un-medicated and the medicated group.

Treatment effects are analyzed applying Multivariate Analysis of Covariance (MANCOVA) with treatment condition as between group factor and PT1 and PT2 scores as within group factor. Pretest scores were entered as covariates.

Speed and quality of text reading did not satisfy criteria for parametric analyses. Differences between baseline and PT1 and PT2 were therefore analyzed with the Mann-Whitney U-test.

## Results

### Baseline Results

The CCPT-II composite measures of Focused Attention and Vigilance were better at baseline among subjects using medication compared to those without medication, whereas the Benton Visual Retention Test was best in the unmedicated group. The average T-scores for Focused Attention in the medicated group were 52 (s.d.9) and 59 (s.d. 13) in the unmedicated group. (F (1,59) = 6.70, p = .012, Eta^2^ = .104). The Vigilance T-scores were 51 (10) and 57 (9) respectively (F (1,59) = 6.07, p = .017, Eta^2^ = .093). Low scores represent a better score. Subjects without medication scored 5.5 (s.d. 1.6) on BVRT, while medicated subjects scored 4.5 (1.8) (F = 5.17, p = .027, Eta^2^ = .078). None of the other NP measures, rating scale measures or academic measures showed group differences related to medication.

The training and control groups did not differ on any measure at pretest. As shown in Table 2, the CCPT scores for both groups at baseline deviated maximally seven T-scores (0.7 s.d.) from the normative mean. The scores for the sample as a whole were average at baseline also with regard to the CAVLT-II measures. The sample averages in scaled scores were 8.6 in Processing speed (s.d.2.2), 9.3 in Controlled Attention (2.1) and 8.4 in TMT-4 (2.3).

The mean baseline scaled score on Key Math was 7.6 (s.d. 2.1) for the total sample. Transforming Logos scores to normative percentiles to give an impression of pretraining reading proficiency showed a mean reading fluency and reading speed on the 25th and 32^nd^-percentile levels, respectively.

The average ADHD-Rating Scale Home version score was 32 (s.d.10) for the boys and 30 (s.d.10) for the girls. This is equivalent to the 93rd percentile cut-off for boys and beyond that level for girls. On the School version, the equivalent figures were 24 (s.d. 11) for the boys and 15 (s.d. 9) for the girls, which is less than 1 s.d. above normal performance for both genders.

Average score on the General Executive Composite (GEC) on the BRIEF was T-score 68 (s.d. 10) for boys and 69 (s.d. 8) for girls in the Home version (i.e.1.8 and 1.9 s.d. above mean respectively). The equivalent figures were 70 and 66 on the School version (s.d. 11 and 12).

The SDQ Total Difficulties scores at home were 17 for boys and 18 for girls (s.d. 6 and 5); the scores on the teacher ratings forms were 13 for boys and 9 for girls (s.d. 6). Ratings from 14 to 16 are considered borderline results.

### Training Effects

The NP test performance at baseline, PT1 and PT2, is shown in Table 1. MANCOVA showed a significant group effect on Processing Speed, while separate ANCOVAs showed that this was significant only at PT1 after controlling for baseline results (Eta^2^ = .105). There were no other significant group differences for any other NP measures.

**Table 1:** Neuropsychological Test performance.

	Pretest		Posttest I		Posttest II		MANCOVA
	Training	Controls	Training	Controls	Training	Controls	*F*
CPT-II^1^ Focus	52 (9)	57 (11)	53 (9)	56 (11)	51 (8)	54 (9)	.86
CPT-II Hyperactivity-Impulsivity	45 (5)	46 (7)	45 (5)	47 (6)	47 (7)	45 (7)	1.23
CPT-II Sustained	51 (10)	52 (13)	50 (13)	51 (11)	51 (11)	50 (11)	.10
CPT-II Vigilance	51 (10)	51 (11)	50 (8)	54 (11)	52 (12)	54 (12)	.92
Processing speed^2^	8.1 (2.0)	9.1 (2.2)	10.2 (1.7)	10.4 (1.8)	9.6 (1.8)*	11.2 (2.3)	1.21
TMT-4^3^	7.5 (3.6)	7.7 (3.9)	8.9 (3.1)	9.3 (3.6)	9.3 (3.2)	9.4 (2.0)	3.67*
CW Controlled attention^4^	9.0 (2.2.)	9.7 (1.9)	10.8 (2.0)	10.9 (1.9)	11.5 (2.1)	11.3 (1.8)	1.28
CAVLT-2^5^ Level of learning	31 (7)	31 (7)	36 (7)	34 (8)	35 (8)	36 (7)	2.03
CAVLT-2 Delayed Recall	9.5 (4.0)	9.8 (3.6)	11.3 (3.3)	11.1(3.3)	10.6 (3.7)	11.4 (2.9)	1.20
CAVLT-2 Recognition	13.4 (2.4)	13.9 (2.2)	14.2 (2.1)	14.2 (2.2)	13.5 (3.5)	14.7 (1.8)	1.28
BVRT^6^	4.7 (1.8)	5.1 (1.7)	5.7 (2.2.)	5.4 (1.9)	5.4 (2.0)	5.4 (2.0)	.90

* p<.05.

CPT-II^1^: Conners’ Continuous Performance Test-version 2; Processing speed^ 2^: mean scaled score of Color Word –color naming, word reading and Trail Making test task 2 and 3; TMT-4^3^: D-KEFS Trail Making Test, task 4 (divided attention); CW Controlled attention^4^: mean scaled score of time and errors of Color Word Interference and Inhibition/swithching; CAVLT-2 ^5^: Children’s Auditory Verbal Learning Test- version 2; BVRT^6^: Benton Visual Retention Test.

Table 2 shows the results on the tests of academic performance. When comparing improvement from pretest to PT1 and from pretest to PT2, the Mann-Whitney U-test showed a group difference both for speed and quality of Text Reading. MANCOVA showed a group difference on the composite measure for Word Decoding Quality, and ANCOVA showed that the Training-group performed better than the control group both at PT1 (Eta^2^ = .337) and PT2 (Eta^2^ = .355) after controlling for pretest-performance.

Table 3 and Table 4 show the parent’s and teacher’s rating scale results. The MANCOVAs showed no significant group effects on any measure.

**Table 2:** Mathematics and Reading performance.

	Pretest		Posttest I		Posttest II		MANCOVA	
	Training	Controls	Training	Controls	Training	Controls	*F*	*p*
Mathematics	7.6 (2.2)	7.6 (2.1)	8.4 (2.6)	7.8 (1.9)	8.2 (2.3)	7.7 (2.4)	1.25	n.s.
LOGOSReading Fluency, % correct	92 (6)	94 (7)	96 (5)**	95 (5)	98 (3**)	96 (4)	Pre-PT1∶812† **Pre-PT2∶719† **	<.001<.001
LOGOSReading Fluency, Time (min.)	5.9 (5.1)	5.1 (4.4)	4.5 (2.3)*	5.7 (9.5)	3.8 (1.5)*	3.8 (1.7)	Pre-PT1∶383† *Pre-PT2∶344† *	.035.016
Word decoding speed	1.42 (.41)	1.36 (.63)	1.37 (.51)	1.14 (.23)	1.10 (.20)	.96 (.17)	1.45	
Word decoding quality (% correct)	74 (16)	79 (12)	85 (11)**	82 (10)	90 (7)**	86 (7)	23.35**	<.001

* *p*<.05,

** *p*<.0.001

† Mann Whitney U.

**Table 3:** Parent Ratings.

	Pretest		Posttest I		Posttest II		MANCOVA
	Training	Controls	Training	Controls	Training	Controls	*F*
ARS-IV^1^: Attention	18.6 (4.3)	17.0 (5.6)	15.0 (5.6)	16.2 (6.2)	15.3 (5.3)	16.5 (5.6)	3.04
Hyperactivity-Impulsivity	14.0 (6.1)	13.4 (7.1)	10.5 (7.2)	11.5 (7.0)	11.6 (6.7)	11.8 (6.2)	.65
Total Score	32.7 (9.0)	30.5 (11.6)	25.2 (11.5)	27.6 (12.3)	27.0 (11.5)	28.1 (11.0)	1.79
SDQ-^2^ Overall	17.7 (6.0)	16.8 (5.8)	15.6 (7.9)	16.0 (7.1)	15.1 (7.5)	16.6 (6.5)	2.00
Impact	4.6 (2.2)	4.1 (2.2)	3.7 (2.7)	4.0 (2.6)	3.8 (2.6)	4.0 (2.9)	.57
BRIEF^3^ Metacognition Index	70 (7)	67 (10)	66 (9)	65 (9)	67 (8)	64 (10)	.77
General Exec.Composite	70 (9)	67 (10)	66 (11)	66 (10)	67 (11)	65 (12)	.86

ARS-IV^1^: ADHD Rating Scale.

SDQ^2^: Strenghts & Difficulties Questionnaire.

BRIEF^3^: Behavior Rating Inventory of Executive Function.

**Table 4:** Teacher Ratings.

	Pretest		Posttest I		Posttest II		MANCOVA
	Training	Controls	Training	Controls	Training	Controls	*F*
ARS-IV^1^: Attention	13.8 (6.1)	13.3 (5.9)	12.4 (6.6)	13.8 (6.8)	13.2 (6.0)	14.5 (6.7)	2.08
Hyperactivity-Impulsivity	8.3 (6.2)	8.0 (6.4)	7.5 (5.4)	8.1 (6.6.)	6.9 (4.8)	8.2 (6.7)	1.60
Total Score	22.1 (11.6)	21.3 (10.7)	19.9 (11.6)	21.9 (12.1)	20.1 (9.8)	22.6 (12.3)	1.99
SDQ^2^–Overall	11.4 (6.6)	12.0 (5.9)	11.3 (7.0)	12.4 (6.0)	10.0 (6.3)	12.4 (4.9)	2.00
Impact	2.5 (1.7)	2.5 (1.8)	1.9 (1.9)	2.8 (2.2)	2.1. (1.9)	2.8 (1.8)	2.80
BRIEF^3^ Metacognition Index	69 (11)	69 (11)	68 (12)	69 (11)	68 (9)	69 (13)	.62
General Exec.Composite	69 (12)	69 (11)	68 (14)	69 (13)	67 (10)	69 (13)	.16

ARS-IV^1^: ADHD Rating Scale.

SDQ^2^: Strenghts & Difficulties Questionnaire.

BRIEF^3^: Behavior Rating Inventory of Executive Function.

## Discussion

Previous research has shown increased performance on WM measures subsequent to training, but transfer effects have been less documented. Analyses of near transfer effects from the same study that are in process of being published have shown differentially increased performance on traditional measures of WM capacity. In the present far transfer analyses, we ask whether this improvement transfers to other cognitive and behavioral domains that would be expected to increase if WM indeed has been improved. The composite measure of processing speed showed a significant improvement in the training group, while no other NP measures showed improvements. However, reading skills improved differentially in the training group. Rating scales showed no significant group differences due to training.

Overall, the present study deviates from previous ones by being the first study reporting predominantly negative findings with regard to transfer of WM training effects to other functional domains. Two previous studies have included a mixture of medicated and unmedicated subjects [8], [25]. Could the use of medication have exhausted the possibility for further improvement? At baseline, medicated subjects performed better with regard to Focused attention and upheld arousal even when not continuously active, evident from the Interstimulus-effect on CPT-II. Although there were no significant differences in IQ between medicated and unmedicated subjects, it is nevertheless possible that children referred for medication are more impaired. This could be the reason for the somewhat surprising finding that the unmedicated subjects performed better on BVRT than their medicated counterparts. With these exceptions, there were no other medication related differences, thus allowing us to include a mixture of them in the two groups. Hypothetically, the lack of improvement on CCPT-II could be because a majority of subjects performed normal already at pretest due to medication. Nevertheless, the participants still were slightly impaired in controlled attention and processing speed, giving room for improvement. The rating scale performance of the subjects at baseline showed that their parents rated them as highly symptomatic, whereas the teachers rated them as generally in the normal range. The BRIEF results were equivalent to the study of Beck and colleagues, which found significant improvement at home but not at school. In the first study on WM-training, Klingberg and colleagues [8] examined a small group of children with ADHD, as well as four adult university students without a WM deficit. The authors drew the conclusion that impairment in WM was not necessary for attaining a training effect since the adult students also improved performance after training. The remaining studies of WM training have either recruited participants on the basis of WM impairments [19] or not given information as to the functional level of their ADHD participants. This makes it difficult to assess the impact of pre-training level. When faced with such modest training effects as presented here, future research on prerequisites for training effect is necessary. Medication has to be controlled, whereas in the present study medication was prescribed based on clinical considerations. It might be that impaired WM is not necessary for transfer of treatment effects, but that subjects who already have optimized their behavior due to medication will show less treatment effects, regardless of whether their pre-training performance was average or below norm.

The initial Klingberg studies [8], [9] found that WM training improved both Stroop Color Word and visual reasoning immediately after completed training. The first Klingberg study [8] also showed improved reaction time. Løhaugen et al. [10] found increased memory. The present study corroborates the Klingberg et al. [8] finding of increased processing speed, but not the Løhaugen finding of increased memory. While Klingberg found a large effect, we find a small effect that was only significant immediately after completed training.

Klingberg et al [8] found a training effect in simple reaction time, but not in choice reaction time and no reduction in RT variability. They were not able to replicate the positive finding in their later study [9]. When considering chronometric measures in ADHD, one has to take into account the speed accuracy trade-off. Timed tests demanding perfect performance, i.e. where errors will result in longer time used since the error must be corrected, can measure effectiveness. Simple reaction time, regardless of errors, however, is often faster in ADHD than among controls [42] reflecting hyperactivity-impulsivity rather than effectiveness. In the current study, reaction time was faster than the normative mean, and did not change with retesting or training. It would not be a training goal to reduce reaction time, but instead to improve effectiveness. The improved processing speed in the composite score based on the less attention-demanding parts of the Color Word and Trail Making tests indicates such an increase in effectiveness.

Regarding memory, we found no effect whatsoever in verbal or visual delayed recall. The reason for the discrepant findings in the current study compared to Løhaugen et al. [10] is probably that they compared pre-post performance in the training groups rather than comparing improvement in training to retest effects in control subjects. Since BVRT measures immediate retention, without necessitating long term storage, it could in fact be considered a test of working memory, albeit different in design from typical span measures or manipulation measures. That the latter showed an effect (unpublished data), while the former did not, suggest that near transfer effects are task specific rather than reflect improved function in WM.

It is well known that NP tests are less sensitive to the symptoms of ADHD subjects [43] compared to rating scales. Nevertheless, in the present study, none of the rating scales showed any significant treatment effects. The BRIEF measures specifically targeting behavior expected to improve from training did not show any overall effect either at school or at home. The study of Beck et al. [25] is comparable to the present in that participants differed about two standard deviations from norms at pretest and remained impaired at school after training. The Beck et al. [25] study, however, differs from the current study with regard to the parent’s scores on the BRIEF. While they found a large effect size with regard to most measures, we found no significant differences, and the direction of the insignificant changes was both positive and negative. In the Beck et al. study, the training was monitored by the parents at home, while in our study, the training was performed at school.

While the rating scales were not convincing with regard to transfer effects of working memory gains, the results in reading were more compelling. Text reading became faster and more correct. Decoding of single words became more correct, although not faster. Dahlin [16] found that a training-mediated increase in WM was a better predictor of improved reading comprehension but not word decoding or orthographic verification. Holmes et al. [17] found no effect of word identification, but an increase in mathematical problem solving. We found that the composite Key Math measure was not significantly increased.

There are some limitations to the study. We have already mentioned that control of medication was not part of the study design, except in the sense that medication should not be changed during the study period. Requiring participants to discontinue effective medication for research purposes for almost one year would most likely not be permitted by the ethical committee approving research projects in Norway.

Another limitation is that the study is not blinded. Teachers, parents and test-administrators knew who were in the training group. The possibility of a double blinded study was ruled out early in the planning phase. The schools were expected to not allocate scarce teacher resources to administer a dummy training condition, that was not expected to show any benefit for their students. Keeping the testing staff blinded to group membership represents a logistical challenge, but should have been done. Regardless, any bias among otherwise professional testing staff would unlikely lead to the pattern of successful near transfer gains and mainly negative findings with regard to far transfer as reported in this study.

Assessing the children with a comprehensive battery of NP and academic tests as well as rating scales, we had to deal with the risk of committing a Type I error if we analyzed all measures separately. As an example, four of 26 attention measures were significant at the five percent level when analyzed separately. On the other hand, we risk committing a Type II error if we corrected for multiple comparisons with a strict Bonferroni correction. Instead we chose to compute robust composite measures, following up the suggestion by Shipstead et al. [21] that several measures of a function must converge to convince us that *the function* has increased. Analyzing both PT1 and PT2 results together in MANCOVAs, and then following up with ANCOVAs only those effects that were significant at this overall level, also contributes to raising the threshold for significance.

Differences in statistical methods compared to previous studies could be one reason for the many insignificant findings in this study compared to the earlier studies. Most previous studies have compared treatment effects in the training and the control groups by separate t-tests or ANOVA for each group. A significant pre-post change in the treatment group only could be simply a reflection of the combination of a retest effect and a genuine training effect. The Beck et al. study represents a methodological exception.

In the evolution of new treatment methods, it is not surprising that early published papers on the treatment method report positive findings. Before the method becomes generally accepted, critical studies must show possible limitations or shortcomings and test the impact of treatment gains. As mentioned, the analyses of near transfer based on the same subjects have shown increased working memory capacity after training and is thus in line with most other studies in concluding that it is possible to increase scores on working memory tests after training. The contribution of the present far transfer analyses, however, is the sobriety with regard to the transfer effect on everyday functioning. Since it is well documented that working memory is important for learning and for attention in daily life, we have to question whether the increase in WM tests reflects an increase in *function*, and instead conclude that increase in near transfer to a large degree represents a narrow training effect on a *task.* We use the modifier “to a large degree” since there were also positive findings with regard to processing speed and reading effectiveness. However, we agree with Melby-Lervåg and Hulme [12] that this may not be sufficient justification for WM training, since such effects can also be achieved by direct training of reading skills. Further research is necessary to improve the quality of the training program and examining conditions or thresholds for success. The claim by Klingberg et al. [8] that WM could be increased regardless of initial impairment seems insubstantiated. By looking for differential effects among subjects with large initial impairments, it is all the more important that care is taken to control for task related and measurement error, as well as retest effects as one would expect a significant regression to the mean.

## Supporting Information

Checklist S1 CONSORT Checklist
(DOC)

Protocol S1 Trial Protocol (English Translation)
(DOC)

Protocol S2 Trial Protocol (Norwegian Translation)
(DOC)
