# Supplementary material for: Few Effects of Far Transfer of Working Memory Training in ADHD: A Randomized Controlled Trial
Source: PLoS One. 2013 Oct 4;8(10):e75660. doi: 10.1371/journal.pone.0075660 (PMC3790857; doi:10.1371/journal.pone.0075660)
Supplement: Protocol S1 — Trial Protocol (English Translation) (DOC) [file pone.0075660.s002.doc]

# PROJECT DESCRIPTION

***Translation from the original Norwegian.***

*ADHD – A study of the effects of working memory training*

Anne Kristine Aarlien, Brit Kari Saunes and Jens Egeland

Appendix 1

Content:

**1. WORKING TITLE** [**3**](#__RefHeading___Toc220995585)

**1.1** **BACKGROUND, TOPIC AND OBJECTIVES** [3](#__RefHeading___Toc220995586)

**1.2** **ISSUES TO BE ADDRESSED** [5](#__RefHeading___Toc220995587)

**1.3** **RESEARCH PLAN, DESIGN AND METHOD** [6](#__RefHeading___Toc220995588)

**1.4** **ETHICAL CONSIDERATIONS for PROJECT** [8](#__RefHeading___Toc220995589)

**1.5** **TIMEFRAME** [10](#__RefHeading___Toc220995590)

SOURCES: [11](#__RefHeading___Toc220995591)

# 1. WORKING TITLE

ADHD – A study of the effects of working memory training.

The research topic is whether computer-based working memory training has an effect on working memory function and whether any effects persist. The target group for the study is children with ADHD. The objective of the study is to gain scientific documentation about a possible treatment form for children with ADHD.

## **BACKGROUND, TOPIC AND OBJECTIVES**

**Empirical research**

For many years investigators have been researching to find out about the role of working memory in cognition. Traditionally, working memory capacity has been viewed as static and fixed.

In two separate experiments using functional MR, Torkel Klingberg, professor of cognitive neuroscience at the Karolinska Institute, has found that working memory training increases activity in the frontal and parietal cortex when patients perform working memory tasks. «These results show that the neural system associated with working memory is plastic, i.e. is amenable». Klingberg describes how the brain works when working memory is active: «Studies have shown that the frontal cortex (frontal lobe) is essential. Other important brain structures are the parietal cortex (parietal lobe) and the basal ganglia. Another important factor is that the neurotransmitter dopamine must be functioning correctly» (Klingberg, 2008).

Together with colleauges, Klingberg has developed a PC-based program for training working memory. He is the founder of Cogmed Systems AB. The program lasts 20 days. The children work daily five days a week for 30 - 45 minutes on training tasks on the computer at home. Assessments conducted in two controlled and randomized studies showed that the training effect was significant at three months follow-up (Klingberg et al., 2005; Westerberg et al., 2007, in Klingberg 2008). There is an uncertainty about the long-term effects, however, because many participants did not complete the training program and it was difficult to keep the control group «blinded» and untreated over time. A school study conducted by Dahlin et al. showed that a significant improvement on tests measuring reading comprehension and mathematical problem solving ability persisted six months after completing training. This study -- in addition to two other studies – seem to indicate that working memory training has long-term effects (Klingberg 2008). Academic institutions in the USA and Europe have also gained an interest in working memory training. Completed and on-going studies are being documented. There are no on-going studies being documented in Norway (Klingberg, 2008).

**Theoretical framework**

A condition that in recent years has received attention relating to working memory impairment is Attention Deficit Hyperactivity Disorder (ADHD) (Klingberg 2008). The diagnostic criteria in ICD-10 commonly used in the child psychiatric services in Norway is F90.0 Hyperkinetic disorder (ADHD). «Attention deficit/hyperactivity disorder (ADHD) is defined as age – inapproriate behavior, with symtoms of inattention, impulsivity and hyperactivity» (American Psychiatric Association, 1994).

Working memory is a basic cognitive function subserving many complex mental tasks. Disturbances in working memory functioning will have major consequences for the individual child’s functioning with regard to general development and academic performance. Working memory is used on a daily basis to remember instructions and in problem-solving, and is defined thus: “Working memory (WM) is the ability to keep information online during a short period of time and is thought to underlie a wide range of mental activities, such as reading, arithmetic and problem solving”. Different authors define the concept of working memory differently. (Baddley and Hitch1974, Desimone 1996, Awh & Jones 2001, Cowan 2001, in Klingberg 2008). Recently completed studies indicate that children with working memory deficits have difficulty focusing on relevant information and tasks, and are more prone to day-dreaming (Kane et al. 2003, in Klingberg 2008).

The Norwegian Health Directorate (2008) has issued official guidelines for psychiatric outpatient clinics working with children and adolescents. The guidelines provide recommendations regarding developing the outpatient clinics’ range of psychiatric services available for children and adolescents. In section 3.15 of a chapter about research and scientific development, the following is written: «The regional health services institutions are responsible for important research activity. It is important that efforts are made to facilitate research in the psychiatric health services field for children and adolescents». In a chapter about therapeutic treatment (section 3.5), the following is written: «The outpatient clinics should design their range of treatment alternatives in line with scientifically documented effective forms of treatment...». The study will be based on the health institutions’ annual plans and central guidelines issued by The Norwegian Health Directorate.

The Norwegian Health Directorate (2007) has issued official guidelines regarding diagnosing and treating ADHD. In principle, the official guidelines can be viewed as recommendations and advice, and shall be guiding for related activities in mental health institutions. The official guidelines mention working memory training under a chapter on other treatment forms in section 5.4 as follows: «The training of memory function (working memory) has been tested in controlled studies and has shown promising results for children with ADHD. Additional research, however, is needed to study the long-term effects, the transfer value to daily living situations and the effect when combined with medicines.».

Working memory training is a treatment method that directly targets an assumed underlying cause of the symptoms for children with ADHD (Klingberg 2008). Measures that are described in the official guidelines for school, home and nursery school and other arenas are aimed at symptoms that are evident in the children’s behavior. Operationalizing and carrying out the measures at school and at home may vary and the effect of the measures may be difficult to measure. There is a need for scientifically based treatment measures for children with ADHD, in which the reason for the symptoms may be treated and the effect of the measures applied may be measured. A great deal of interest has been expressed in working memory training from psychiatric outpatient clinics in Vestfold.

## **ISSUES TO BE ADDRESSED**

The main issue to be addressed in the study is to what degree training with the computerized training program from Cogmed improves working memory function in children with the diagnosis of ADHD in the age group 10-12 years.

Specifically we ask the following:

-Will a training group perform better than control group at the end of the training?

-Will any training effect persist over time, i.e. also at retesting six months after completed training?

-Will the training effect be evident in various types of measures of working memory function, i.e. neuropsychological tests and report forms assessing working memory function in daily life at home and at school.

-Specificity:

-Will any effects from working memory training be specific to working memory function, or provide a generally improved cognitive function? Correlations will be examined between improvements in working memory and measures of other cognitive functions, as well as to measures of academic performance.

## **RESEARCH PLAN, DESIGN AND METHOD**

**Organisation of project**

The project is a collaborative effort between the the Departments for Child and Adolescent Psychiatry in Vestfold and Telemark Hospitals and the Research Unit at Vestfold Hospital Trust (PIV HF). The project is organized with a steering group consisting of team leader/psychologist Brit Kari Saunes, ST, educational therapist Anne-Kristine Aarlien, BUPA, team leader Bodil Sjømæling in the Neuropsychiatric team BUPA-Vestfold and research manager Jens Egeland, PIV HF. In addition, a person with administrative or research compentency from ST will also join the group.

Locally in the two hospitals, two project groups have been established consisting of staff linked to implementing the project. In Vestfold, this project group is the Neuropsychiatric team at BUPA. Two persons will be appointed from each outpatient clinic in the county who have attended coach training and will assist in carrying out the training program. In Telemark, the project will be organized by the Neuroteam at the Child and Youth clinic. The team is administratively placed under the Specialized Treatment section for children and adolescents, but has a county-wide responsibility for providing neuropsychiatric functional assessments for patients who normally receive treatment from the child and youth psychiatric outpatient clinics.

**Design**

The experimental design involves a pretest and posttests with a control group.

Post-test 1 will take place after completion of the training program. Post-test 2 will take place six months later.

**Planned selection of participants/sources**

Patients who have received the diagnosis Hyperkinetic disorder/ADHD in Vestfold or Telemark will be asked to participate when they – as a normal part of the clinical activity – contact their local outpatient clinic or neuroteam. After information is provided to parents and the child, and a consent form is signed by the parents, a drawing will be held to determine assignment to intervention or control group. If the intervention (computer training) has an effect, the participants in the control group will also be able to participate in computer training after the study is completed. The goal is to recruit at least 25 participants for an intervention group and 25 participants for a control group from Vestfold, and 20 children from Telemark. The control group will receive “treatment as usual”. The intervention group will also continue to receive any other ongoing treatment (medication, measures at school and at home) as before, in addition to the working memory training.

Children with co-occuring conditions such as serious psychiatric disorders (bipolar disorder), mild mental retardation, co-occuring Aspergers syndrome/autism spectrum disorders, epilepsi or Tourettes syndrome will be excluded from participating in the study.

Children with ADHD have differing symptomotology depending on gender. Males and females will be included, but a precondition for participation is that they have an ADHD combined type diagnosis, which includes both attention problems, hyperactivity and impulsivity. In order to reduce the age-related variance in the relevant variables to be examined, the study will be limited to children 10-12 years of age.

**Instruments**

To ensure construct validity, the working memory construct must be operationalized. There must be a consistent relationship between the construct and the empirical indicators. A limited selection of standardized and normed neuropsychological and academic-related tests designed to measure working memory functions will be used.

It is important to have several measures of working memory and that the measures vary in modality. It will be relevant to apply neuropsychological tests of working memory such as the following: Trailmaking tests from D-KEFS, Letter-number, and the children’s version of Paced Auditory and Serial Addition Test (PASAT). In order to measure whether any effect of training is specific for working memory and not a general cognitive improvement, it will also be relevant to analyze tests of other cognitive functions that are part of a standard neuropsychological test battery used by the hospital’s neuroteam, such as the Children’s Auditory Verbal Learning Test (CAVLT-2) and (individual tasks) from the IQ test Wechlser Abbreviated Scale of Intelligence (WASI). In addition, academic-related measures will be applied, with subtests from LOGOS and KEYMATH (reading and mathematics test batteries).

In addition to standardized neuropsychological and academic-related tests, parent-, school- and self-report forms of behavior will be administered at pretest and post-tests. It is suggested to use the questionnaire BRIEF (Behavior rating inventory of executive function). This is a behavior rating form for executive functions in which working memory is one component. BRIEF is standardized and normed in English, with an approved Norwegian translation that has been tested for equivalent psychometric properties in a methodological study conducted by psychologist Øyvind Fallmyr at BUPA. SDQ will also be administered.

**Collection of data/carrying out study**

Participants in the intervention group and control group will be assessed with the neuropsychological and academic-related tests and behavior rating scales prior to training and approximately ½ year after completed training. The intervention group will also be assessed two weeks after completed training. The start of training will begin as soon as the recruitment process for the project is completed.

The computer training program lasts five weeks and involves daily training sessions (5 weekdays) lasting approx. one school hour. The training will be administered by a teacher at the child’s school and integrated into the normal school day.

During the training period, there will be telephone contact between the project group and the teacher on a weekly basis regarding status of the training.

When assessing the study’s validity overall, Cook and Cambell’s (1979) validity system will be used. The validity system includes statistical validity, internal validity, construct validity and external validity.

**Analysis of data material**

Students t-test for independent samples and Variance analysis will be used for significance testing. The statistical program SPSS will be used for statistical analyses.

## **ETHICAL CONSIDERATIONS FOR PROJECT**

Research ethics encompass a wide diversity of values, norms and institutional arrangements that contribute to forming and regulating scientific activity. In all scientific work, strict requirements are set for research ethical considerations. NESH (Research ethical guidelines for the social and human sciences, law and theology) describe the overall ethical guidelines.

All children in the study under 12 years and their parents/guardians must consent to their child’s participation in the project. A prerequisite is informed and free consent (NESH, section 9), and the child’s own acceptance is necessary from the age when they are old enough to express such an acceptance (NESH, section 12). A secure system must be in place so that all data is anonymous, and that the data cannot be traced to individuals (NESH, sections14, 16). Children and parents/guardians must be informed of the effect of the intervention. There is a requirement of informing about results to the participants in an understandable form (NESH, section 47). The plan for the study will be sent to the Regional Committee for Medical and Health Research Ethics, Health Region South East (REK). A notification will also be sent to the Norwegian Social Science Data Service (NSD), (NESH, section 10).

I do not see any major ethical issues linked to the working memory training study. Participants will likely see a self-interest in participating in the study. Children are usually easily motivated to use computers.

## **TIMEFRAME**

| Summer 09 | Approval by department head, University of Oslo, Regional Ethical Committee and the Norwegian Social Science Data Service. Meetings with research unit at PiV. |
| --- | --- |
| June 09 | Coach course in working memory training for leaders in the project group. |
| August-December 09 | Pretest. Start of working memory training. Post-test one. Writing/Data analyses. |
| March- October 2010  ­­­­­­­ | Post-test two  ­­­­­ |
| October 10 | Control group receives offer of training |
| October 10→ May 11 | Write/data analyses. Presentation of results. |

References:

American Psychiatric Association, (1994), *Diagnostic and statistical manual of mental disorders* (4th ed.). Washington, DC: Author.

Barkley, RA (1997), *Behavioral inhibition, sustained attention, and executive functions: constructing a unifying theory of ADHD.* Psychol Bull 121, 65-94.

Klingberg, T (2008), *Träning av arbetsminnet (Training of Working Memory)* – Karolinska institutet. Stockholm.

Klingberg, Torkel (2008), *The concept of working memory.*  Karolinska institute, Stockholm.

Klingberg, Torkel (2008), *Genomförd forskning (Completed research). Pågående forskning (Ongoing research).*

Kleven, T A 2002, `Begrepsoperasjonalisering` (Construct operationalization), in Lund, T. (ed.), *Innføring i forskningsmetodologi (Introduction to resesarch methodology),* Unipub Forlag, Oslo, s. 141 – 182.

Kvernbekk, T 2002, `Vitenskapsteoretiske perspektiver` (Scientific perspectives), in Lund, T. (ed.), *Innføring i forskningsmetodologi (Introduction to resesarch methodology),* Unipub Forlag, Oslo, s. 19 – 73.

Lund, T 2002, `Metodologiske prinsipper og referanserammer` (Methodological principles and frames of reference), in Lund, T (ed.), *Innføring i forskningsmetodologi (Introduction to resesarch methodology),* Unipub Forlag, Oslo, s. 79 - 121.

NESH (ed.) (2006), *Forskningsetiske retningslinjer for samfunnsvitenskap, humanoria, juss og teologi (Research ethical guidelines for the social and human sciences, law and theology),* Oslo: Forskningsetiske kriterier.

*Veileder i diagnostikk og behandling av AD/HD (Official guidelines for diagnosing and treatment of AD/HD),* Sosial og helsedirektoratet (Norwegian Directorate of Health), 2007.

*Veileder for poliklinikker i psykisk helsevern for barn og unge (Official guidelines for outpatient clinics in the psychiatric health services sector for children and adolescents*, Helsedirektoratet (Norwegian Directorate of Health) 2008.

Westerberg H, Hirvikoski T, Forssberg H, & Klingberg T (2004), *Visuo-spatial working memory: a sensitive measurement of cognitive deficits in ADHD.* Child Neuropsychology.
