# Supplementary material for: Few Effects of Far Transfer of Working Memory Training in ADHD: A Randomized Controlled Trial
Source: PLoS One. 2013 Oct 4;8(10):e75660. doi: 10.1371/journal.pone.0075660 (PMC3790857; doi:10.1371/journal.pone.0075660)
Supplement: Protocol S2 — Trial Protocol (Norwegian Translation) (DOC) [file pone.0075660.s003.doc]

# PROSJEKTBESKRIVELSE

*ADHD - En effektstudie av arbeidsminnetrening*

Anne Kristine Aarlien, Brit Kari Saunes og Jens Egeland

Vedlegg 1

Innholdfortegnelse:

**1. ARBEIDSTITTEL** [**3**](#__RefHeading___Toc220995585)

**1.1** **BAKGRUNN, TEMA OG FORMÅL** [3](#__RefHeading___Toc220995586)

**1.2** **PROBLEMSTILLING** [5](#__RefHeading___Toc220995587)

**1.3** **FORSKNINGSOPPLEGG, DESIGN OG METODE** [6](#__RefHeading___Toc220995588)

**1.4** **ETISKE HENSYN SOM MÅ IVARETAS I PROSJEKTET** [8](#__RefHeading___Toc220995589)

**1.5** **TIDSPLAN** [10](#__RefHeading___Toc220995590)

Kildeliste: [11](#__RefHeading___Toc220995591)

# 1. ARBEIDSTITTEL

ADHD – En effektstudie av arbeidsminnetrening.

Forskningsspørsmålet er om databasert arbeidsminnetrening har effekt på arbeidsminnets funksjon og om effekten opprettholdes. Målgruppen for studien er barn med ADHD. Hensikten med studien er vitenskapelig dokumentasjon på en mulig egnet behandlingsform for barn med ADHD.

## **BAKGRUNN, TEMA OG FORMÅL**

**Empirisk forskning**

Det har i flere tiår vært forsket på å finne ut hvordan hjernen fungerer når man bruker arbeidsminnet. Arbeidsminnets kapasitet har tradisjonelt blitt sett på som statisk og uforanderlig.

Torkel Klingberg, professor i kognitiv nevrovitenskap ved det Karolinska institutt, har ved to ulike eksperiment ved bruk av funksjonell MR funnet ut at arbeidsminnetrening øker aktiviteten i frontalcoretex og parietalcoretex når pasienten utfører arbeidsminneoppgaver. «Dessa resultat visar att de neurale system som är assosierade till arbeidsminnet är plastiska dvs. har förandringsbara». Klingberg beskriver hvordan hjernen fungerer når man bruker arbeidsminnet: «Studier har visat att frontalcortex (panneloben) är mycket viktig. Andra viktiga strukturer är perietalcortex (hjässloben) och de basale ganglierne. Det är også viktigt att signalisering med transmittorsubstansen dopamin fungerar korrekt» (Klingberg, 2008).

Klingberg har sammen med sine medarbeidere utarbeidet et databasert program for trening av arbeidsminnet. Han er grunnleggeren av Cogmed Systems AB. Programmet strekker seg over 20 dager. Barna arbeider daglig 5 dager i uken i 30 - 45 minutter med treningsoppgaver på PC i hjemmet. Målinger utført ved to kontrollerte og randomiserte studier viste at treningseffektene var signifikante etter en tre måneders oppfølging (Klingberg et al., 2005; Westerberg et al., 2007, i Klingberg 2008). Det er imidlertid knyttet usikkerhet rundt langtidseffektene, fordi deltagerne har hatt frafall og det har vært vanskelig og beholde kontrollgruppen «blind» og ubehandlet over tid. En skolestudie gjennomført av Dahlin et al., viste at det fortsatt var signifikant forbedring på test som måler leseforståelse og matematisk problemløsningsevne seks måneder etter avsluttet trening. Denne studien samt to andre studier indikerer at arbeidsminnetrening har en langidseffekt (Klingberg 2008). Akademiske institusjoner i USA og Europa har også fattet interesse for arbeidsminnetreningen. Det dokumenteres gjennomførte og pågående studier. Det dokumenteres ikke pågående studier i Norge (Klingberg, 2008).

**Teoretisk ramme**

En tilstand som man i de senere år har blitt oppmerksom på når det gjelder arbeidsminneproblem er Attention Deficit Hyperactivity Disorder (ADHD) (Klingberg 2008). Diagnosekriteriene i ICD - 10 som vanligvis brukes i barnepsykiatrien i Norge er F90.0 Hyperkinetisk forstyrrelse (ADHD). «Attention deficit/hyperactivity disorder (ADHD) is defines as age – inapproriate behavior, with symtoms of inattention, impulsivity and hyperactivity» (American Psychiatric Association, 1994).

Arbeidsminnet er en grunnleggende kognitiv funksjon som ligger til grunn for mange mentale komplekse oppgaver. Forstyrrelse av arbeidsminnet vil ha har store konsekvenser for det enkelte barns funksjon med hensyn til generell utvikling og akademisk ytelse. Arbeidsminnet brukes i hverdagen til å huske instruksjoner, hva vi skal gjøre for å løse problemer, kontrollere oppmerksomhet, og huske hva vi skal konsentrere oss om. Barkley (1997) definerer arbeidsminnet slik: “Working memory (WM) is the ability to keep information online during a short period of time and is thought to underlie a wide range of mental activities, such as reading, arithmetic and problem solving”. Ulike forfattere definerer begrepet arbeidsminet ulikt. (Baddley and Hitch1974, Desimone 1996, Awh & Jones 2001, Cowan 2001, i Klingberg 2008). Nyere gjennomførte studier tyder på at barn med et redusert arbeidsminne har vansker med å fokusere på relevant informasjon og oppgaver og bedriver mer dagdrømmeri (Kane et al. 2003, i Klingberg 2008).

Helsedirektoratet (2008) har gitt ut en veileder for poliklinikker i psykisk helsevern for barn og unge. Veilederen gir føringer for videreutvikling av poliklinikkenes tilbud innen psykisk helsevern for barn og unge. I kapittel om forskning og fagutvikling punkt 3.15 står det: « De regionale helseforetakene står for en viktig forskningsaktivitet. Det er viktig at det legges til rette for forskning i det psykiske helsevesenet for barn og unge». Under kapittel om behandling punkt 3.5 står det: « Poliklinikkene bør utforme sine behandlingstilbud i tråd med vitenskapelig dokumentasjon om virkningsfulle behandlingsformer...». Det søkes å forankre studien i virksomhetens årsplaner og sentrale føringer fra Helsedirektoratet.

Sosial- og helsedirektoratet (2007) har gitt ut en veileder i diagnostikk og behandling av ADHD. Veilederen er i prinsippet å regne som anbefalinger og råd og skal være styrende for virksomheten. Veilederen omtaler arbeidsminnetrening under kapittel om annen behandling punkt 5.4. slik: «Trening av hukommelsesfunksjonen (arbeidsminne) utprøvd i kontrollerte studier har vist lovende resultater for barn med ADHD. Ytterligere forskning er imidlertid nødvendig for å studere langtidseffektene, overføringsverdiene til dagliglivets situasjoner og effekten av kombinasjonen med legemidler».

Arbeidsminnetrening er en behandlingsmetode som retter seg direkte mot en antatt årsak til symptomene for barn med ADHD (Klingberg 2008). Tiltak som er beskrevet i veilederen for skole, hjem og barnehage og andre arenaer retter seg mot symptomene som vises i barnas atferd. Operasjonaliseringen og gjennomføringen av tiltakene i skole og hjem kan være varierende og effekten av tiltakene kan synes vanskelig å måle. Det understrekes et behov for vitenskapsbaserte behandlingsmetoder for barn med ADHD der årsaken til symptomene kan behandles og effekten av tiltakene som settes inn kan måles. Det meldes om stor interesse for arbeidsminnetrening fra poliklinikkene i psykiatrien i Vestfold.

## **PROBLEMSTILLING**

Det overordnede spørsmålet i undersøkelsen er i hvilken grad trening med det komputeriserte treningsprogrammet fra Cogmed bedrer arbeidsminnefunksjonen hos barn med diagnosen ADHD i alderen 10-12.

Konkret spør vi om:

-Vil treningsgruppen prestere bedre enn kontrollgruppen ved avslutning av treningen?

-Vil en eventuell treningseffekt holde seg over tid, dvs. også ved retesting seks måneder etter avsluttet trening.

-Vil treningseffekten være synlig på ulike typer mål på arbeidsminnefunksjon. Dvs. nevropsykologiske funksjonsmål og kartlegging av arbeidsminnefunksjon i dagliglivet hjemme og på skolen.

-Spesifisitet:

-Vil en effekt av arbeidsminnetrening være spesifikk for arbeidsminnefunksjonen eller gi en generell bedret kognitiv funksjon? Korrelasjoner mellom bedring av arbeidsminne og mål på andre kognitive funksjoner, samt på skolefaglig fungering.

## **FORSKNINGSOPPLEGG, DESIGN OG METODE**

**Organisering av prosjektet**

Prosjektet er et samarbeid mellom Barne og Ungdomsklinikken, Sykehuset Telemark, Barne og Ungdompsykiatrisk Avdeling (BUPA) i Psykiatrien i Vestfold (PIV HF) og Forskingsenheten i PIV HF. Prosjektet er organisert med en styringsgruppe bestående av teamleder/psykolog Brit Kari Saunes, ST, klinisk pedagog Anne-Kristine Aarlien, BUPA, teamleder Bodil Sjømæling i Nevropsykiatrisk team BUPA-Vestfold og forskningssjef Jens Egeland, PIV HF. I tillegg vil en person med administrativt eller forskningsansvar fra ST, tiltre gruppen.

Lokalt i de to sykehusene er det organisert to prosjektgrupper av personale som er knyttet til gjennomføringen av prosjektet. I Vestfold er det Nevropsykiatrisk team ved BUPA. Det oppnevnes to behandlere fra hver poliklinikk i fylket som har gjennomgått coachutdanningen og som skal bistå med gjennomføringen av treningen. I Telemark organiseres prosjektet fra nevroteamet i Barne- og ungdomsklinikken. Teamet er administrativt plassert inn under Habiliteringsseksjon for barn og unge, men har et fylkesansvar for å gi nevropsykologiske funksjonsutredninger for pasienter som vanligvis får et behandlingstilbud fra barne- og ungdomspsykiatriske poliklinikker.

**Design**

Det planlegges et eksperimentelt design, pretest og posttest med kontrollgruppe.

Posttest en utføres etter gjennomkjørt treningsprogram. Posttest to gjennomføres etter seks måneder.

**Planlagt utvalg av informanter/kilder**

Pasienter som hav fått diagnosen Hyperkinetisk forstyrrelse/ADHD i Vestfold eller Telemark vil bli forespurt om deltagelse når de som del av ordinær klinisk virksomhet har kontakt med sin lokale poliklinikk eller nevroteam. Etter at informasjon er gitt til foreldre og barn, og informert samtykke er underskrevet av foreldrene, vil det bli foretatt en loddtrekning om hvem som skal delta i eksperiment og kontrollgruppen. Hvis intervensjonen (datatreningen) gir effekt, vil også deltagerne i kontrollgruppen få tilbud om datatrening etter at forskningsprosjektet er avsluttet. Man tar sikte på å rekruttere minimum 25 deltagere i behandlingsgruppen og 25 deltagere i kontrollgruppen fra Vestfold samt 20 barn fra Telemark. Kontrollgruppen vil motta ”treatment as usual”. Også i eksperimentgruppen vil den øvrige behandling (medikasjon, tiltak på skole og i hjem) fortsette som før, med tillegg av arbeidsminnetreningen.

Barn med komorbide tilstander som alvorlig psykiatriske lidelse (bipolar lidelse), mild mental retardasjon, komorbid Aspergers syndrom/autismespektrumforstyrrelser, epilepsi eller Tourettes syndrom utelukkes i studien.

Barn med ADHD kan ha ulik symptomatologi på kjønn. Gutter og jenter inkluderes, men det forutsettes ADHD kombinert type, som inkluderer både oppmerksomhetsvansker, hyperaktivitet og impulsivitet. For å redusere aldersrelatert varians i de aktuelle variabler som skal undersøkes, vil studien avgrenses til å gjelde barn fra 10-12 år.

**Instrumenter**

For å sikre begrepsvaliditet må arbeidsminnebegrepet operasjonaliseres. Det må være samsvar mellom begrep og empirisk indikator. Det gjøres et avgrenset utvalg av standardiserte og normerte nevropsykologiske og spesialpedagogiske tester som er konstruert til å måle arbeidsminnefunksjoner.

Det er viktig å ha flere mål på arbeidsminne og at målene varierer i modalitet. Det vil være aktuelt å anvende nevropsykologiske tester av arbeidsminne som: Trail-making testene fra D-KEFS, tallspenn baklengs, og barneversjon av Paced Auditory og Serial Addition Test (PASAT). For å måle at en eventuell treningseffekt som er spesifikk for arbeidsminnet og ikke en generell kognitiv forbedring, vil det også være aktuelt å analysere tester av andre kognitive funksjoner som inngår i det standard nevropsykologiske testbatteriet som Nevropsykiatrisk team anvender, så som Children’s Auditory Verbal Learning Test (CAVLT-2) og (delprøver) fra evnetesten Wechlser Abbreviated Scale of Intelligence (WASI). I tilegg vil det benyttes spesialpedagogiske verktøy med deltester fra LOGOS og KEYMATH (språk og matematikk).

I tilegg til standardiserte nevropsykologiske og spesialpedagogiske tester, vil det vurderes om det skal brukes foreldre, skole og selv registreringsskjema av atferd ved pretest og posttest. Her foreslås å bruke spørreskjema BRIEF (Behavior rating inventory of executive funktion). Dette er et kartleggingsskjema for styringsfunksjoner der arbeidsminne inngår som en komponent. BRIEF er standardisert og normert på engelsk, men en godkjent norsk oversettelse har vært testet for hvorvidt den norske versjonen har samme psykometriske egenskaper i et metodeprosjekt i regi av psykolog Øyvind Fallmyr ved BUPA. I tillegg vil SDQ bli benyttet.

**Innsamling av data/gjennomføring av studien**

Deltagerne i eksperimentgruppen og kontrollgruppen undersøkes med de nevropsykologiske og spesialpedagogiske testene og kartleggingsskjemaene før treningen og ca ½ år etter avsluttet trening. Eksperimentgruppen undersøkes også 2 uker etter avsluttet trening. Igangsetting av trening vil foretas fortløpende ettersom pasienter rekrutteres inn i prosjektet.

Datatreningsprogrammet strekker seg over fem uker og innebærer daglig trening (5 ukedager) over ca en skoletime. Treningen vil gjøres av lærere ved barnets skole og mest mulig integreres i den

Det vil under treningen være telefonkontakt mellom prosjektgruppen og lærer ukentlig vedrørende gjennomføringen av treningen.

Ved den totale vurderingen av undersøkelsens validitet vil Cook og Cambell (1979) sitt validitetssystem bli brukt. Validitetstssystemet inkluderer statistisk validitet, indre validitet, begrepsvaliditet og ytre validitet.

**Analyse av datamaterialet**

Det brukes Students t-test for uavhengige utvalg og Variansanalyse for signifikanstesting. For den statistiske behandlingen brukes statistikkprogrammet SPSS.

## **ETISKE HENSYN SOM MÅ IVARETAS I PROSJEKTET**

Forskningsetikk viser til et mangfold sett av verdier, normer og institusjonelle ordninger som bidrar til å konstituere og regulere vitenskapelig virksomhet. I alt vitenskapelig arbeid stilles det strenge krav til forskningsetiske hensyn. NESH (Forskningsetiske retningslinjer for

samfunnsvitenskap, humanoria, juss og teologi) beskriver de overordnede etiske retningslinjer.

Alle barn i studien er under 12 år, og foreldre/foresatte må gi sitt samtykke til deres barns deltagelse i prosjektet. Det forutsettes informert og fritt samtykke (NESH, punkt 9), og barnets egen aksept er nødvendig fra de er gamle nok til å uttrykke den (NESH, punkt 12). Det må sikres at alle data anonymiseres og at data ikke kan spores til personer (NESH, punkt14, 16). Barn og foreldre/foresatte informeres om effekten av tiltaket. Det er krav om å tilbakeføre resultater til deltagerne i en forståelig form. (NESH, punkt 47). Planen for studien sendes Regional komité for medisinsk forskningsetikk Helseregion sørøst (REK). Det sendes meldning til Norsk samfunnsvitenskapelig datatjeneste (NSD), (NESH, punkt 10).

Jeg kan ikke se store etiske problemstillinger knyttet til gjennomføringen av arbeidsminnetreningen. Utvalget vil trolig kunne se en egeninteresse for deltagelse. Barn motiveres vanligvis lett av dataverktøy.

## **TIDSPLAN**

| sommer 09 | Godkjenning av avdelingssjef, UIO, REK og NSD. Møte med forskningsenheten i PiV. |
| --- | --- |
| Juni 09 | Coach kurs i arbeidsminnetrening for lederne av prosjektgruppen. . |
| august-desember 09 | Pretest. Igangsetting av arbeidsminnetrening. Posttest en. Skrive/Databearbeiding. |
| Mars- Oktober 2010  ­­­­­­­ | Posttest to  ­­­­­ |
| Oktober 10 | Kontrollgruppen får tilbud om datatrening |
| Oktober 10→ mai 11 | Skrive/databearbeiding. Presentasjon av resultater. |

Kildeliste:

American Psychiatric Association, (1994), *Diagnostic and statistical manual of mental disorders* (4th ed.). Washington, DC: Author.

Barkley, RA (1997), *Behavioral inhibition, sustained attention, and executive functions: constructing a unifying theory of ADHD.* Psychol Bull 121, 65-94.

Klingberg, T (2008), *Träning av arbetsminnet* – Karolinska institutet. Stockholm.

Klingberg, Torkel (2008), *The consept of working memory.*  Karolinska institute, Stockholm.

Klingberg, Torkel (2008), *Genomförd forskning. Pågående forskning.*

Kleven, T A 2002, `Begrepsoperasjonalisering`, i Lund, T. (red.), *Innføring i forskningsmetodologi,* Unipub Forlag, Oslo, s. 141 – 182.

Kvernbekk, T 2002, `Vitenskapsteoretiske perspektiver`, i Lund, T. (red.), *Innføring i forskningsmetodologi,* Unipub Forlag, Oslo, s. 19 – 73.

Lund, T 2002, `Metodologiske prinsipper og referanserammer`, i Lund, T (red.), *Innføring i forskningsmetodologi,* Unipub Forlag, Oslo, s. 79 - 121.

NESH (red) (2006), *Forskningsetiske retningslinjer for samfunnsvitenskap, humanoria, juss og teologi,* Oslo: Forskningsetiske kriterier.

*Veileder i diagnostikk og behandling av AD/HD,* Sosial og helsedirektoratet, 2007.

*Veileder for poliklinikker i psykisk helsevern for barn og unge*, Helsedirektoratet 2008.

Westerberg H, Hirvikoski T, Forssberg H, Klingberg T (2004), *Visuo-spatial working memory: a sensitive measurement of cognitive deficits in ADHD.* Child Neuropsychology.
